# Macrophage AMPK β1 activation by PF-06409577 reduces the inflammatory response, cholesterol synthesis, and atherosclerosis in mice

**DOI:** 10.1016/j.isci.2023.108269

**Published:** 2023-10-20

**Authors:** Emily A. Day, Logan K. Townsend, Sonia Rehal, Battsetseg Batchuluun, Dongdong Wang, Marisa R. Morrow, Rachel Lu, Lucie Lundenberg, Jessie H. Lu, Eric M. Desjardins, Tyler K.T. Smith, Amogelang R. Raphenya, Andrew G. McArthur, Morgan D. Fullerton, Gregory R. Steinberg

**Affiliations:** 1 Centre for Metabolism, Obesity and Diabetes Research, Department of Medicine, McMaster University, Hamilton, ON, Canada; 2 Division of Endocrinology and Metabolism, Department of Medicine, McMaster University, ON, Canada; 3 Department of Biochemistry and Biomedical Sciences, McMaster University, Hamilton, ON, Canada; 4 Department of Biochemistry, Microbiology and Immunology, Faculty of Medicine, Centre for Infection, Immunity and Inflammation, Ottawa Institute of Systems Biology, Centre for Catalysis Research and Innovation, University of Ottawa, Ottawa, ON, Canada

**Keywords:** Immunology

## Abstract

Atherosclerotic cardiovascular disease is characterized by both chronic low-grade inflammation and dyslipidemia. The AMP-activated protein kinase (AMPK) inhibits cholesterol synthesis and dampens inflammation but whether pharmacological activation reduces atherosclerosis is equivocal. In the current study, we found that the orally bioavailable and highly selective activator of AMPKβ1 complexes, PF-06409577, reduced atherosclerosis in two mouse models in a myeloid-derived AMPKβ1 dependent manner, suggesting a critical role for macrophages. In bone marrow-derived macrophages (BMDMs), PF-06409577 dose dependently activated AMPK as indicated by increased phosphorylation of downstream substrates ULK1 and acetyl-CoA carboxylase (ACC), which are important for autophagy and fatty acid oxidation/*de novo* lipogenesis, respectively. Treatment of BMDMs with PF-06409577 suppressed fatty acid and cholesterol synthesis and transcripts related to the inflammatory response while increasing transcripts important for autophagy through AMPKβ1. These data indicate that pharmacologically targeting macrophage AMPKβ1 may be a promising strategy for reducing atherosclerosis.

## Highlights

• AMPKβ1 specific activator PF-06409577 potently activates AMPK in macrophages
• PF-06409577 reduces atherosclerosis via myeloid AMPK β1 in mice
• AMPK activation by PF-06409577 reduces NF-κB and inflammatory pathways in macrophages

## Graphical abstract

**Figure:**
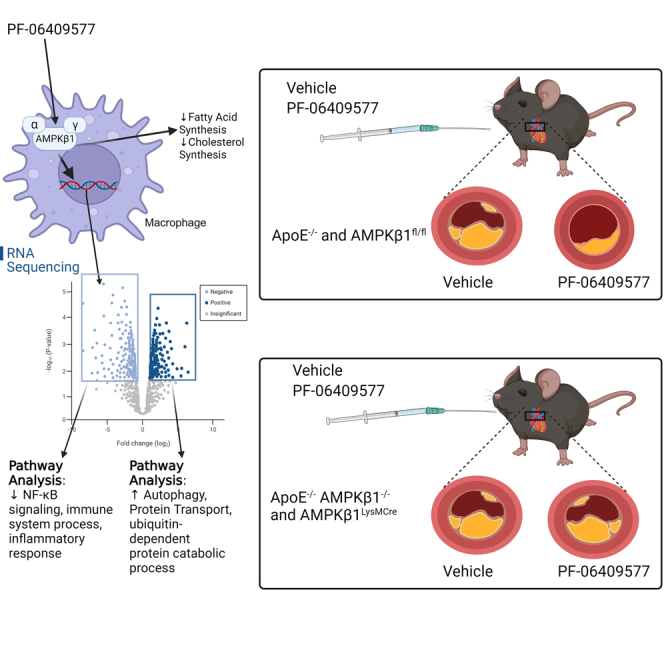

## Introduction

Atherosclerotic cardiovascular disease is one of the leading causes of death worldwide. Risk factors for atherosclerotic disease include high levels of low-density lipoprotein (LDL) cholesterol, and triglyceride rich lipoproteins (TGRL).^1^^,^^2^ In addition to hyperlipidemia, inflammation, including both the innate and adaptive immune system, are fundamental drivers of atherosclerosis.^3^^,^^4^ Atherosclerotic plaques are rich in both cholesterol and immune cells, specifically macrophages, resulting in the accumulation of lipid laden macrophages (also referred to as foam cells) within the intima of large blood vessels. These macrophage rich atherosclerotic plaques can be further characterized by lipid accumulation, inflammatory cytokine release, recruitment of additional immune cells, cellular death (apoptosis or necrosis) and fibrosis within the arterial walls.

The AMP-activated protein kinase (AMPK) is a ubiquitously expressed heterotrimeric protein that consists of an α, β, and γ subunit. The activation of AMPK reduces the synthesis of fatty acids and cholesterol, inflammation and cell proliferation while simultaneously enhancing fatty acid oxidation, mitochondrial homeostasis, and autophagy^5^^,^^6^^,^^7^^,^^8^^,^^9^^,^^10^; pathways which are dysregulated in atherosclerotic cardiovascular disease.^11^^,^^12^^,^^13^^,^^14^^,^^15^ AMPK is activated by a wide range of small molecules and xenobiotics that disrupt mitochondrial function leading to increases in AMP/ADP (i.e., berberine, metformin, and canagliflozin) or mimic the effects of AMP/ADP (i.e., AICAR) (for more details see reviews by Day et al.^16^ and Steinberg et al.^17^). However, given increases in AMP/ADP have a wide array of effects on multiple metabolic enzymes and many compounds were developed with a focus on alternative metabolic targets (i.e., SGLT2 inhibition) some of the beneficial effects of these agents to reduce atherosclerosis are likely mediated through pathways not requiring AMPK.^18^^,^^19^^,^^20^^,^^21^ Adding to the uncertainty about the role of AMPK in atherosclerosis is that the effects of genetically removing various AMPK subunits on atherosclerosis are equivocal with some studies showing no effect, increases, or reduced atherosclerosis.^5^^,^^14^^,^^21^^,^^22^^,^^23^^,^^24^

Over the last decade, several small molecules which directly bind to the regulatory AMPKβ1 isoform and allosterically activate the kinase without disrupting mitochondrial function have been developed for treating metabolic diseases including type 2 diabetes, metabolic associated fatty liver disease (MAFLD, formerly known as NAFLD) and autosomal dominant polycystic kidney disease (ADPKD) (for more details see reviews by Day et al.^16^ and Steinberg et al.^17^). The β1 subunit of AMPK is highly expressed in the liver, adipose tissue, and immune cells. AMPK β1 is critical for suppressing lipid synthesis in the hepatocytes and macrophage inflammation and increasing fatty acid oxidation in both hepatocytes and macrophages,^5^^,^^21^^,^^25^^,^^26^^,^^27^ suggesting that targeting this subunit may be beneficial for cardiometabolic diseases such as SLD and atherosclerosis.

PF-06409577 is an orally bioavailable, indole carboxylic acid, that selectively binds to the allosteric drug and metabolite (AdaM) site of the AMPK β1 isoform, leading to allosteric activation with an EC50 of approximately 3.3 nM with limited off target effects.^28^^,^^29^ In contrast to previous β1 selective AMPK activators such as A769662, PF-06409577 is more potent and has more favorable pharmacokinetic properties for oral administration, in rats, dogs, and monkeys.^28^ Specifically, dose response studies in mice indicated that maximal inhibition of liver *de novo* lipogenesis occurred with oral delivery in methylcellulose at 100 mg/kg, a dose which corresponded with serum concentrations of ∼8 nM, and is consistent with the EC50 for maximally activating AMPKβ1 complexes in cell free assays.^29^ And while previous studies have shown that treatment of mice and non-human primates with PF-06409577 lowers liver steatosis and serum cholesterol^29^ the effects of targeting the AMPK β1 subunit with PF-06409577 in macrophages and mouse models of atherosclerosis is currently unknown.

In the current study we find that PF-06409577 reduces atherosclerosis in ApoE^−/−^ and PCSK9 over-expression mouse models through a pathway requiring the AMPK β1 isoform. Surprisingly, these reductions in atherosclerosis were not associated with changes in hepatic or serum lipid levels. Instead, we found that PF-06409577 induced reductions in atherosclerosis required AMPK β1 in myeloid cells and this dose dependently activated AMPK in bone marrow-derived macrophages (BMDMs) reducing cholesterol and fatty acid synthesis, lowering the expression of inflammatory response and I-kappaB kinase/NF-κB regulated transcripts and increasing the expression of transcripts important for autophagy. These data suggest that activating AMPK β1 complexes in macrophages may be important for reducing atherosclerosis.

## Results

### PF-064095777 reduces atherosclerosis via AMPK β1 in ApoE^−/−^ mice independently of reductions in circulating triglycerides or cholesterol

**Figure 1:**
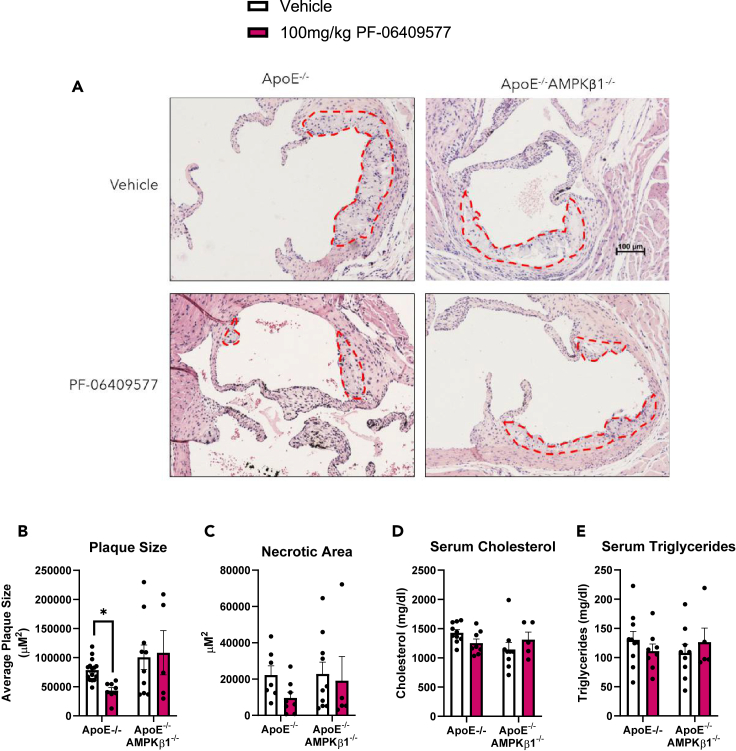
PF-064095777 reduces Atherosclerosis via AMPK β1 in ApoE^-/-^ mice independently of reductions in circulating triglycerides or cholesterol ApoE^−/−^ and ApoE^−/−^ AMPK β1^−/−^ mice were fed a Western diet and treated with vehicle or 100 mg/kg PF-06409577 by oral gavage. (A) Representative images of plaques in the aortic root, with plaques outlined in red, scale bar represents 100 μM, (B) plaque quantification, (C) necrotic area, (D) serum cholesterol, and (E) triglycerides. Data are presented as mean ± SEM, ∗ indicates p < 0.05 by two-way ANOVA with Fisher’s LSD post-hoc testing.

Whole body ApoE^−/−^ or ApoE^−/−^ AMPKβ1^−/−^ mice were fed a Western diet and treated daily with PF-06409577. PF-06409577 did not alter body mass, adiposity, lean mass, or insulin sensitivity in ApoE^−/−^ or ApoE^−/−^ AMPKβ1^−/−^ mice over the 6-week treatment period (Figures S1A–S1E) while having a very minor effect on improving glucose tolerance (Figures S1F and S1G). Despite similar adiposity and insulin sensitivity, PF-06409577 reduced atherosclerotic plaques size by ∼ 45% in the aortic root of ApoE^−/−^ but not ApoE^−/−^ AMPKβ1^−/−^ mice (Figures 1A and 1B). Similarly, although not significant, PF-06409577 treatment tended to reduce necrotic area within the plaques of ApoE^−/−^ but not ApoE^−/−^ AMPKβ1^−/−^ (Figure 1C). In contrast to observations in hyperlipidemic rats or non-human primates where PF-06409577 reduced serum triglycerides and cholesterol, surprisingly there were no effects on these parameters in ApoE null mice (Figures 1D and 1E). Consistent with the lack of liver steatosis and inflammation in ApoE mice there was no change in liver lipids or the mRNA expression of markers of liver monocyte or macrophage infiltration (*F4/80*) or inflammatory cytokines (*Tnfα* or *Il1b*) following treatment with PF-06409577 in either genotype (Figures S2A–S2E). These data indicate that PF-06409577 reduces atherosclerosis in ApoE null mice through a mechanism requiring AMPKβ1 but is independent of reductions in serum cholesterol in this model.

### PF-06409577 induced reductions in atherosclerosis involves myeloid AMPKβ1

**Figure 2:**
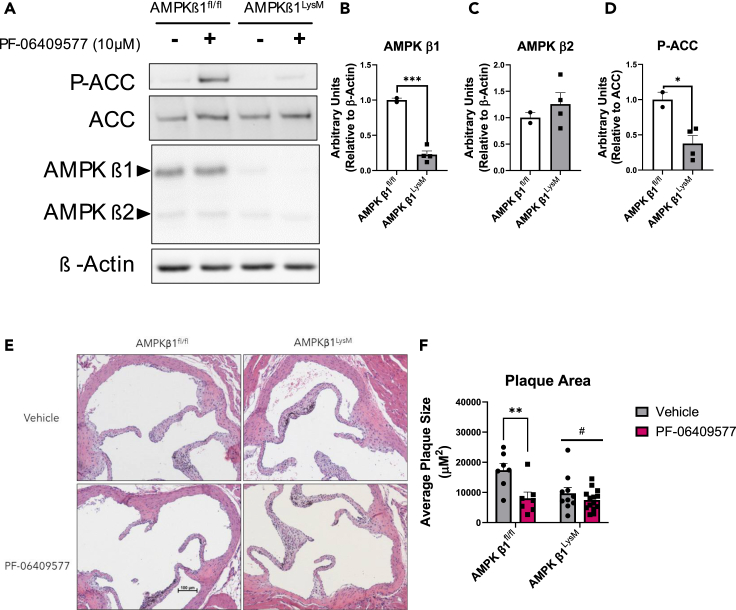
PF-06409577 reduces atherosclerosis via macrophage AMPK activation (A–D) Peritoneal macrophages were isolated from AMPK β1^fl/fl^ and AMPK β1^LysM^ mice. (A) AMPK levels and phosphorylation of ACC following treatment with PF-06409577 (10 μM, 90 min) were detected by western blot and (B–D) quantified using ImageJ data are presented as mean ± SEM, ∗ indicates p < 0.05 and ∗∗∗ indicates <0.005 by unpaired t test. AMPK β1^fl/fl^ and AMPK β1 ^LysM^ mice were injected with PCSK9 AAV, fed a Western diet and treated with Vehicle or 100 mg/kg PF-06409577 by oral gavage for 6 weeks. (E and F) Representative plaque images, scale bar represents 100 μM and quantification. Data are presented as mean ± SEM, # indicates p < 0.05 in overall group effect my two-way ANOVA, ∗ indicates p < 0.05 by two-way ANOVA with Fisher’s LSD post-hoc testing.

Given serum cholesterol levels were unchanged in the ApoE^−/−^ mice, we hypothesized that PF-06409577 may reduce atherosclerosis by activating AMPKβ1 complexes within immune cells, including macrophages. To test this hypothesis, we crossed mice with Cre-recombinase expression driven by the LysM promoter to mice with the AMPKβ1 allele floxed (hereafter referred to as AMPKβ1^fl/fl^ or AMPKβ1^LysM^) to generate myeloid specific AMPK β1^−/−^ mice. Isolation of peritoneal macrophages showed an ∼80% reduction in AMPKβ1 with no change in AMPKβ2 expression and a greater than 50% reduction in phosphorylation of acetyl-CoA carboxylase (ACC) (Figures 2A–2D), indicating significant reductions in AMPK activity in the macrophage compartment.

To induce atherosclerosis in AMPKβ1^fl/fl^ or AMPKβ1^LysM^ mice, we injected a gain-of-function PCSK9 AAV via the tail vein to over-express PCSK9 in the liver, which leads to reductions in the liver LDLr and increases in serum cholesterol when mice are fed a Western diet.^24^^,^^30^^,^^31^ As anticipated, injection with the PCSK9 AAV resulted in a 20-fold increase in serum PCSK9 in both AMPKβ1^fl/fl^ or AMPKβ1^LysM^ mice (Figure S3A). Daily oral gavage with PF-06409577 resulted in a subtle reduction in body weight in both AMPKβ1^fl/fl^ and AMPKβ1^LysM^ mice compared to vehicle controls (Figure S3B). Treatment with PF-06409577 did not alter serum or liver cholesterol and exerted only modest effects on serum and liver triglycerides (Figures S3C–S3F). Despite similar serum and liver lipid profiles, PF-06409577 reduced atherosclerotic plaque size in AMPKβ1^fl/fl^ but not AMPKβ1^LysM^ mice (Figures 2E and 2F). Consistent with previous studies,^5^ AMPKβ1^LysM^ mice had smaller atherosclerotic plaques than WT controls (Figures 2E and 2F). These data indicate that PF-06409577 reduces atherosclerosis in mice injected with a PCSK9 AAV and that AMPKβ1 in myeloid cells may be important for mediating this effect.

### Treatment of macrophages with PF-06409577 activates AMPK and markers of autophagy while reducing fatty acid and cholesterol synthesis and genes related to the inflammatory response and I-kappaB kinase/NF-κB

**Figure 3:**
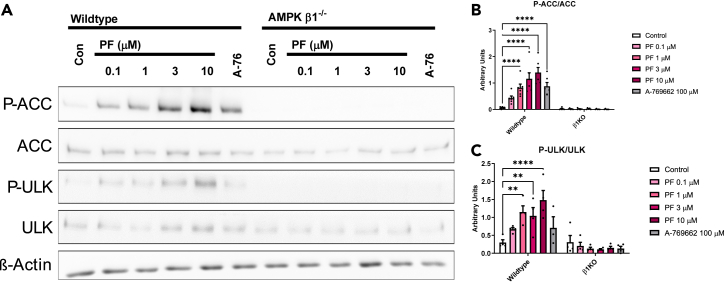
PF-06409577 activates AMPK in macrophages Bone marrow derived macrophages were isolated from wildtype and AMPK β1^−/−^ mice. (A–C) cells were treated with indicated concentrations of PF-06409577(PF) or A-769662(A-76) (100 μM) for 90 min in serum free media. Total and phosphorylation ACC and ULK were measured by immunoblotting. Data are mean ± SEM, ∗ indicates p < 0.05 by two-way ANOVA with Dunnet's post-hoc testing, n = 3.

To evaluate the molecular pathways being affected in macrophages we isolated BMDM from WT and AMPKβ1 null mice and conducted a dose response with PF-06409577. PF-06409577 increased the phosphorylation of downstream targets of AMPK, ACC, and ULK1, following 1.5 h of treatment at 1 μM, 3 μM, and 10 μM doses in WT but not AMPK β1^−/−^ macrophages (Figures 3A–3C). These effects are consistent with previous dose responses with PF-06409577 in murine, rat, monkey, and human hepatocytes.^29^ Additionally, when compared to A-769662 at 100 μM, the first generation AMPKβ1 selective activator,^24^^,^^32^^,^^33^ there was a greater increase in phosphorylation of ACC and ULK1 with just 10 μM of PF-06409577, indicating much greater potency for activating AMPK.

**Figure 4:**
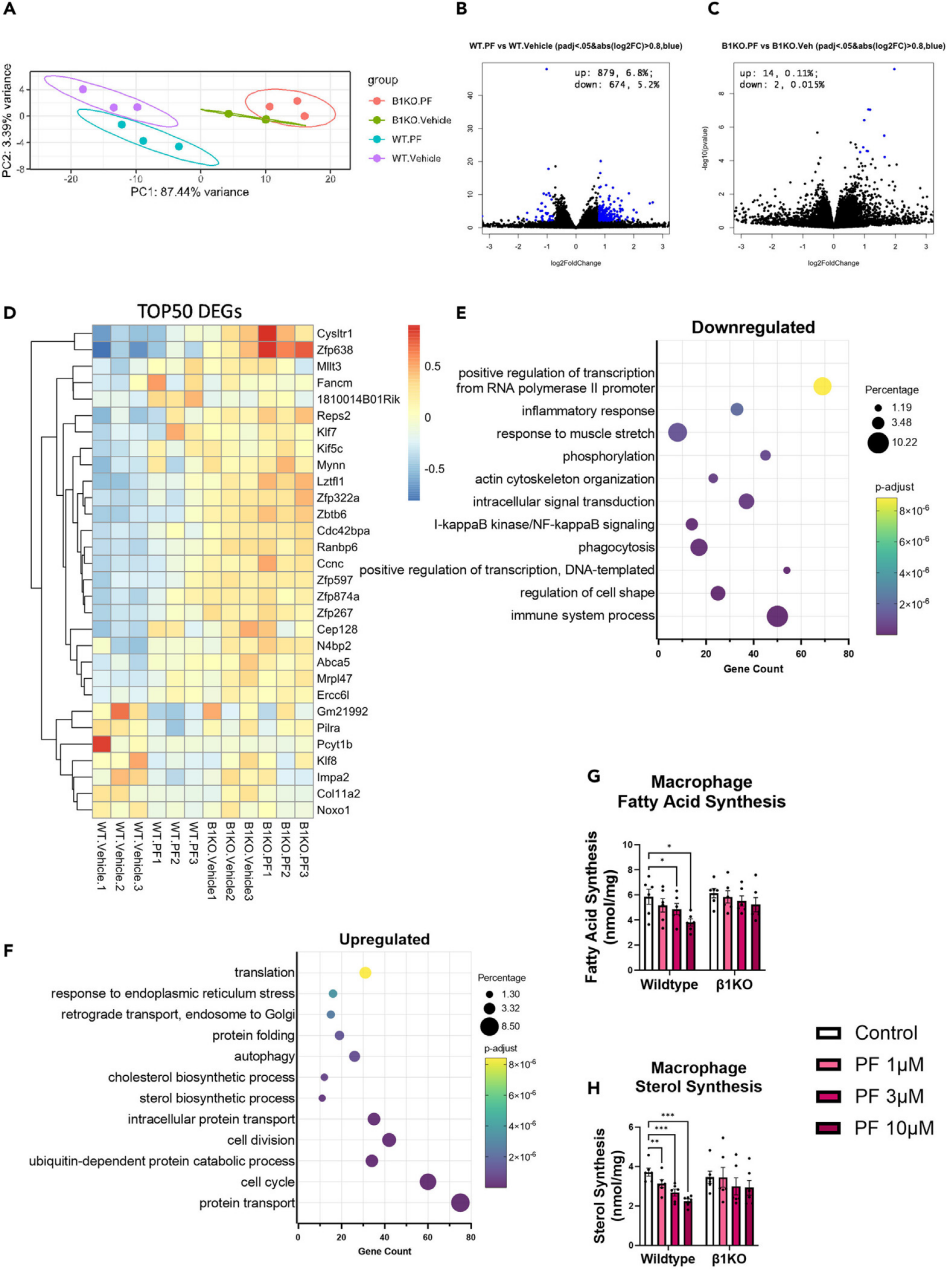
PF-06409577 reduces inflammatory pathways and lipid synthesis in macrophages Wild type and AMPK β1^−/−^ BMDMs were treated with 10 μM PF-06409577 for 6 h and analyzed by RNA-Seq. (A) PCA plots, (B and C) differentially regulated genes in wild type and AMPK β1^−/−^, respectively, (D) heatmap showing log-fold change of the top 50 differentially regulated genes, (E and F) AMPK β1 specific downregulated and upregulated biological process with PF-06409577 treatment. Wild type and AMPK β1^−/−^ BMDMs were treated with 10 μM PF-06409577(PF) for 4 h. (G) Fatty acid and (H) cholesterol synthesis. Data are presented as mean ± SEM, ∗p < 0.05, ∗∗p < 0.01, ∗∗∗p < 0.001, ∗∗∗∗p < 0.0001 by two-way ANOVA with Dunnet's post-hoc testing.

RNA sequencing in WT and AMPKβ1^−/−^ BMDMs indicated that PF-06409577 was highly specific for AMPKβ1, with distinct clustering found by principal component analysis (PCA) analysis (Figure 4A). Further, examination of differentially regulated genes found that in WT mice PF-06409577 resulted in upregulation of 879 genes and downregulation of 674 genes with minimal changes to gene expression in AMPKβ1^−/−^ macrophages (Figures 4B and 4C). When examining the top 50 most differentially expressed genes (DEGs) (Figure 4D), it was notable that several of these genes are involved in regulation of transcription including RNA Pol II, including *Klf8*, *Zfp322a*, and *Klf7*. To gain a more descriptive understanding of the pathways being altered we performed gene ontology analysis of the DEGs. Interestingly, GO terms that were most significantly downregulated included structural elements including regulation of cell shape and actin cytoskeleton organization, but also inflammatory response, immune system processes, phagocytosis and I-kappaB kinase/NF-κB signaling (Figure 4E). Specifically, downregulated transcripts regulated by I-kappaB kinase/NF-κB included *Rela*, also known as p65 of NFkB, and *Nfkb2*, *Tlr9*, *Mapk14*, and *IRF8* (Table S1).

PF-06405977 also led to the AMPK-dependent upregulation of several biological processes including protein transport, and surprisingly an upregulation of catabolic processes including protein catabolic processes and sterol and cholesterol biosynthetic processes and autophagy (Figure 4F). Consistent with AMPK activation of ULK1, PF-06409577 upregulated the expression of genes critical for autophagy including *Atg5*, *Atg12*, and *Lamp2*. Surprisingly, PF-06409577 also upregulated genes in cholesterol synthesis including *Hmgcs*, *Hmgcr*, *Fdft1*, *Cyp51*, and *Insig* (Table S2). PF-06409577 inhibits cholesterol and fatty acid synthesis in hepatocytes suggesting upregulation of cholesterol synthesis genes may have been to compensate for reduced metabolic flux through this pathway. To directly test this hypothesis we assessed fatty acid and sterol synthesis in BMDMs and found that PF-06409577 dose dependently reduced both fatty acid and cholesterol synthesis in an AMPKβ1-dependent manner (Figures 4G and 4H). These data show that PF-06409577 induced reductions in atherosclerosis were associated with the suppression of fatty acid and cholesterol synthesis, induction of markers of autophagy (increased ULK1 phosphorylation and *Atg5*, *Atg12*, and Lamp2) and the suppression of genes related to inflammatory responses, immune system processes, and IkappaB kinase/NF-κB signaling.

## Discussion

In the current study, we show that PF-06409577 reduces atherosclerosis in two mouse models. Further, these effects are dependent on myeloid AMPK β1, as the effects of PF-06409577 on atherosclerosis were absent in both whole body AMPKβ1 knock-out mice and myeloid specific AMPK β1 knockout mice. These data are in contrast to studies with another direct AMPKβ1 activator, A-769662, which failed to reduced atherosclerosis in mouse models,^24^ likely reflects that PF-06409577 is a much more potent activator of AMPK than A-769662 (as shown in Figure 3).

The absence of changes in hepatic and serum lipid levels in the current study is in contrast to effects seen with the same dose of PF-06409577 in C57Bl/6J mouse model fed a high-fat diet as well as studies in non-human primates.^29^ While unexpected, it is potentially related to the hyperlipidemic nature of these atherosclerotic models and alterations to classical LDL clearance, which is consistent with statins typically not yielding robust reductions in atherosclerosis or serum LDL in the ApoE^−/−^ mouse model.^34^ Furthermore, these data demonstrate that extra-hepatic targets can alter atherosclerotic plaque development. ApoE^−/−^ mice have drastically altered lipoprotein metabolism, serum lipid profiles and hepatic lipid levels, compared to other model systems; however, the atherosclerotic plaque morphology seen in this model is most similar to that of humans.^34^ Future studies examining the effects of PF-06409577 in other models of atherosclerosis, including LDL receptor null mice and mini pigs, will be important to confirm whether observations are translatable across distinct models of atherosclerosis.

We conducted a dose response in BMDMs and found that PF-06409577 increased the phosphorylation of two downstream substrates of AMPK, ULK1, and ACC in an AMPK β1-dependent manner. Consistent with the phosphorylation of ACC, PF-06409577 dose dependently reduced fatty acid synthesis and cholesterol synthesis in bone marrow macrophages from WT but not AMPK β1 KO mice. Similarly, consistent with the phosphorylation of ULK1, RNAseq analysis indicated one of the most upregulated GO terms was autophagy. Increases in autophagy and reductions in lipid synthesis have been linked to reductions in macrophage inflammation and atherosclerosis.^11^^,^^12^^,^^13^^,^^14^^,^^15^ Treatment of macrophages with PF-06409577 lowered GO terms related to the inflammatory response, immune system process, phagocytosis and IkappaB kinase/NF-κB signaling. These data indicate that PF-06409577 inhibits lipid synthesis and markers of inflammatory response while promoting markers of autophagy in macrophages suggesting this may be important for the effects of this compound to lower atherosclerosis in mouse models.

In conclusion, this study shows that oral delivery of the small molecule AMPK activator PF-06409577 reduces the development of atherosclerotic plaques in a myeloid AMPKβ1 dependent manner. Furthermore, these reductions in atherosclerosis occurred independently of changes in serum cholesterol. Instead, PF-06409577 activation of AMPK in macrophages reduced transcripts related to the inflammatory response, phagocytosis, immune system processes and IkappaB kinase/NF-κB signaling while suppressing cholesterol and fatty acid synthesis and increasing markers of autophagy. In the setting of cardiometabolic disease where MASLD, insulin resistance and atherosclerosis are commonly linked, these data suggest that an AMPKβ1 specific activator such as PF-06409577 may be effective not only at reducing liver steatosis and insulin resistance but also by targeting macrophages and atherosclerosis. Future studies examining whether the effects of other AMPKβ1 selective compounds which have been shown to have positive effects on MASLD and insulin resistance in mouse models^35^ and phase 2 clinical trials^36^ also have effects on atherosclerosis will be informative to confirm these observations.

### Limitations of study

A limitation of our findings is that we did not complete a dose response examining the effects of PF-06409577 on atherosclerosis. Previous studies have completed *in vivo* dose responses examining the effects of PF-06409577 on increasing AMPK activity in the kidney^28^ and inhibiting liver *de novo* lipogenesis^28^^,^^29^ and found that maximal effects are observed at a dose of 100 mg/kg. Therefore, while we selected this same dose and delivery method of PF-06409577, we did not complete a dose response study examining the effects of PF-06409577 on atherosclerosis or activation of AMPK in macrophages. Therefore, future dose response studies in mice will be important.

## STAR★Methods

### Key resources table

**Table:** 

REAGENT or RESOURCE	SOURCE	IDENTIFIER
**Antibodies**		

Phospho-Acetyl-CoA Carboxylase (Ser79) Antibody	Cell Signaling Technology	Cat# 3661; RRID: AB_330337
ACC	Cell Signaling Technology	Cat# 3662; RRID: AB_2219400
AMPK β1/β2	Cell Signaling Technology	Cat# 4150; RRID: AB_10828832
ULK1(D8H5) Rabbit mAb	Cell Signaling Technology	Cat# 8054; RRID: AB_11178668
Phospho-ULK1 (Ser555) (D1H4) Rabbit mAb	Cell Signaling Technology	Cat# 5869; RRID: AB_10707365
beta-Actin (13E5) Rabbit mAb (HRP Conjugate	Cell Signaling Technology	Cat# 5125; RRID: AB_1903890

**Bacterial and virus strains**		

AAV Virus - AAV8-D377Y-mPSCK9	Vector Biolabs	AAV-268246

**Chemicals, peptides, and recombinant proteins**		

A-769662	LC Laboratories	A-1803-100MG
Acetic acid [3H] sodium salt	Perkin Elmer	NET003005MC

**Critical commercial assays**		

Infinity cholesterol kit	Thermo Scientific	TR13421
Triglyceride Colorimetric Assay Kit	Cayman Chemicals	10010303
PCSK9 ELISA	R&D Systems	MPC900

**Deposited data**		

RNA Seq Data	This paper	GEO: GSE242663
Original Western Blots	This Paper	Mendely Data: https://doi.org/10.17632/k45j97zwbd

**Experimental models: Organisms/strains**		

ApoE Mouse Line	Jackson Laboratories	B6.129P2-Apoetm1Unc/J
LysM Cre Mouse Line	(Banskota et al.^37^)	N/A
AMPK β1KO Mouse line	(Dzamko et al.^25^)	N/A
AMPK β1 flox Mouse line	(Banskota et al.^37^)	N/A

**Oligonucleotides**		

ActB	Thermo Fischer Scientific	Mm00607939_s1
Adgre1(F4/80)	Thermo Fischer Scientific	Mm00802529_m1
IL1b	Thermo Fischer Scientific	Mm00434228_m1
TNFa	Thermo Fischer Scientific	Mm00443258_m1

**Software and algorithms**		

ImageJ		https://imagej.nih.gov/ij/

### Resource availability

#### Lead contact

Further information and requests for experimental details should be directed to the lead contact, Gregory Steinberg (gsteinberg@mcmaster.ca).

#### Materials availability

This study did not generate new unique reagents.

### Experimental model and study participant details

#### Animals

All animal experiments were approved by McMaster University Animal Research Ethics Board. The generation and characterization of AMPKβ1^-/- 25^, ApoE^-/-^AMPKβ1^-/- 14^ mice have been described previously. Myeloid-Specific AMPKβ1 KO mice were generated by crossing AMPK β1flox/flox mice with mice expressing LysM-driven Cre-recombinase as previously described.^37^ Mice were group housed at conventional temperature (22°C–23°C) on a 12-hour light-dark schedule with *ad libitum* access to food and water. Male mice were fed a western Diet (TD.09821, Envigo) for 6 weeks. At the same time as the diet switch, AMPK β1^fl/fl^ and AMPK β1 ^LysM^ mice were injected with 1x10^11^ PCSK9 AAV tail vein injection while anesthetized by isoflurane. One week later, serum PCKS9 levels were measured, and mice were randomized to their treatment groups. All mice were gavaged daily with either vehicle (0.5% Carboymethcellose/0.1% Tween-80) or 100 mg/kg PF-06409577. This dose and delivery method of PF-06409577 was selected based off of previous studies in mice in which the pharmacokinetics of the compound had been carefully examined and where it was shown that a dose of 100 mg/kg elicited serum concentrations of 8nM and exerted maximal effects on lowering liver denovo lipogenesis.^28^^,^^29^ For the glucose and insulin tolerance tests, mice were fasted for 6 hours (beginning at 7 am), basal blood glucose measurements were taken and tests were initiated with an intraperitoneal (i.p) injection of 2g/kg D-glucose or 0.7U/kg insulin, respectively. Blood glucose was measured at the indicated time points using an Accu-Chek Performa blood glucose meter. After 6 weeks mice were anaesthetised with Ketamine (75mg/kg) and Xylazine (10mg/kg) before harvesting tissues.

#### Cell culture

##### Bone marrow derived macrophages (BMDMs)

BMDMs were generated by isolating marrow from the tibia and femur of each leg by centrifugation as described.^38^ Marrow was then suspended in DMEM supplemented with 10% FBS and 1% antibiotic-antimycotic. After 5 hours L929 media (a source of M-CSF) was added and cells were plated in 10cm tissue culture dishes. Cells were then allowed to differentiate for 7 days before reseeding for experiments as described.

##### Peritoneal macrophages

Mice were i.p. injected with 1ml of 10% thioglycolate. On day 4 mice were euthanized and peritoneal cells were collected in PBS containing 5mM EDTA. Cells were washed with DMEM (supplemented with 20% FBS + 1% antibiotic-antimycotic), counted and plated at 1x 10^6^ cells/ml in 6 well plates in DMEM supplemented with 20% FBS and 1% antibiotic-antimycotic. 2-4 hours later cells were washed with DMEM and cultured in DMEM 10% FBS and 1% antibiotic-antimycotic overnight. The following morning media was replaced with serum free DMEM. Cells were treated with 10μM Pf-06409577 for 90 minutes. Cells were snap frozen with Liquid nitrogen and analyzed by immunoblot as described below.

### Method details

#### Atherosclerotic measurements

The heart and aortic root were dissected out, formalin fixed, paraffin embedded and 4 μm thick sections of the aortic root were then collected and stained with H&E to measure plaque size at 80 μm intervals as previously described.^39^^,^^40^ Images were captured using Nikon 90 Eclipse microscope (Nikon). Atherosclerotic plaque size and necrotic area was determined from 3-5 sections per mouse and the area was calculated by manually outlining the plaques on ImageJ software by an individual who was blinded to the groups.

##### Immunoblotting

Following cell lysis, protein content was quantified using the Peirce BCA assay. Lysate protein content was normalized with lysis buffer and cell lysates were diluted with Western sample buffer and loaded in SDS/PAGE gels. Proteins were resolved by molecular mass and transferred to polyvinylidene difluoride membranes prior to blocking in 5% bovine serum albumin. Membranes were incubated with primary antibodies (listed in the key resources table) overnight at 4 degrees. Membranes were washed and incubated in secondary antibody for 1hour at room temperature in 5%BSA. Proteins were detected using Clarity ECL reagent (BioRad).

##### *De Novo* lipogenesis

Lipogenesis methodology was adapted from previous publications.^21^^,^^29^ Cells were reseeded in 12 well plates at 1 × 10^6^ cells/ml and allowed to adhere overnight. The following day media was replaced with serum free DMEM with [^3^H]-acetate and indicated doses of PF-06409577. Cells were washed 3x with ice cold PBS and scraped in KOH (1M in EtOH). Samples were vortexed and then left for 2 h at 70 °C in a shaking incubator. Following samples reaching room temperature, H_2_O and n-hexane were added, vortexed, and samples were centrifuged for 5 min (1500 rpm). Top phase was then added to scintillation vial for counting of the sterol fraction. 2 N HCl and petroleum ether were added to the bottom phase. This solution was vortexed and then centrifuged for 5 min (1500 rpm) at room temperature. The top phase was then added to a scintillation vial for counting of the fatty acid fraction.

##### RNAseq

RNA isolation was done as outlined by manufacturers' specifications. MultiQC was used for quality control of raw data (∼12.5 million paired-end per sample; 50 base pairs each) from RNAseq.^41^ Trim Galore was used to automate quality and adapter trimming as well as quality control. We quantified the expression of transcripts using RNA-seq data through Salmon.^42^ In Salmon’s transcript-level quantifications, DESeq2 was applied for the detection of differentially expressed genes (DEG)^43^ by using the following threshold: |log2(fold change)| > 0.8, adjusted p < 0.05. Principal component analysis (PCA) was performed by using variance stabilizing transformation (VST) data through DESeq2.

DAVID Gene Ontology assessment was used to look at differentially regulated biological processes.^44^^,^^45^ Data is available through GEO database with GEO# GSE242663.

#### ELISAs

Serum PCSK9 levels were analysed using a murine PCSK9 ELISA (Cat# MPC900, R&D Systems) as per the manufacturer’s instructions.

#### Rt-q-PCR

RNA was isolated using RNEasy Columns (Qiagen) with on column DNase treatment, RT-q-PCR was performed as previously described.^21^ All Taqman primers were purchased from Invitrogen, and relative gene expression was calculated using (2–ΔCT) method. Values were normalized to housekeeping gene β-actin to Wildtype Control.

#### Plasma and liver triglycerides and cholesterol

Plasma lipids were measured from mice fasted for 6 hours prior to sacrifice. Blood was collected retro-orbitally through a heparinized capillary tube into an Eppendorf containing EDTA. Samples were diluted 10-fold and cholesterol was measured using the Infinity cholesterol kit (TR13421, Thermo Scientific) according to manufacturer instructions. Triglycerides were measured in samples diluted 2-fold using Triglyceride Colorimetric Assay Kit (10010303, Cayman Chemicals) according to manufacturer instructions. For liver analyses, ∼20 mg of snap frozen liver was homogenized and lipids isolated via the Folch method. The same kits were used to quantify liver triglycerides and total cholesterol which was normalized to tissue weight.

### Quantification and statistical analysis

All statistical analysis was performed using Graphpad Prism version 8. Unless otherwise noted, data were normally distributed and analysed with a two-way ANOVA, with post-hoc analysis performed as indicated in figure captions.

## Data Availability

• Data; RNA-seq data and original western blot images have been deposited in publicly available datasets. DOIs and accession numbers are available in the key resources table. • Code: This paper does not report original code. • All other data reported in this paper will be shared by the lead contact upon reasonable request.

## Supplemental information

Document S1. Figures S1–S3

Table S1. AMPK specific downregulated gene ontologies, related to Figure 4

Table S2. AMPK specific upregulated gene ontologies, related to Figure 4
