# Supplementary material for: Macrophage AMPK β1 activation by PF-06409577 reduces the inflammatory response, cholesterol synthesis, and atherosclerosis in mice
Source: iScience. 2023 Oct 20;26(11):108269. doi: 10.1016/j.isci.2023.108269 (PMC10654588; doi:10.1016/j.isci.2023.108269)
Supplement: Document S1. Figures S1–S3 [file mmc1.pdf]

iScience, Volume *26*

Supplemental information

Macrophage AMPK β1 activation by PF-06409577 reduces the inflammatory response, cholesterol synthesis, and atherosclerosis in mice

Emily A. Day, Logan K. Townsend, Sonia Rehal, Battsetseg Batchuluun, Dongdong Wang, Marisa R. Morrow, Rachel Lu, Lucie Lundenberg, Jessie H. Lu, Eric M. Desjardins, Tyler K.T. Smith, Amogelang R. Raphenya, Andrew G. McArthur, Morgan D. Fullerton, and Gregory R. Steinberg

Supplemental Figure 1

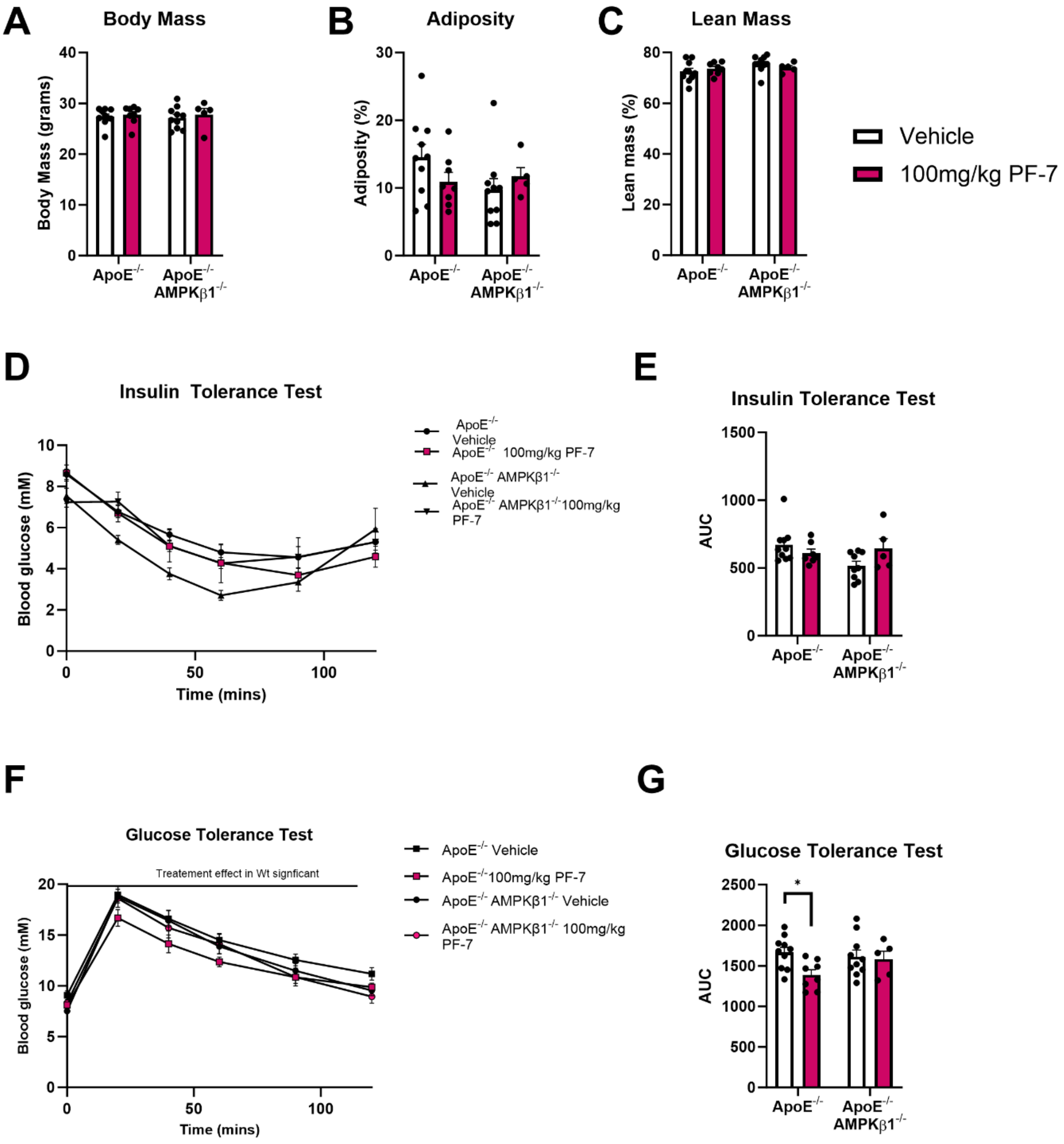

Supplemental Figure 1: PF-06409577 treatment does not alter body mass or body composition. Related to Figure 1 ApoE$^{-/-}$ and ApoE$^{-/-}$ AMPK β1$^{-/-}$ mice were fed a Western diet and treated with vehicle or 100mg/kg PF-06409577 for 6 weeks. **A**) body mass, **B**) adiposity, and **C**) lean mass were measured, **D,E**) insulin tolerance was assessed using 0.7u/kg insulin IP, **F,G**) glucose tolerance was assessed using 2g/kg glucose via ip injection. n=5-10. Data are presented as mean +/- s.e.m., * indicates $p<0.05$ by two-way ANOVA.

Supplemental Figure 2

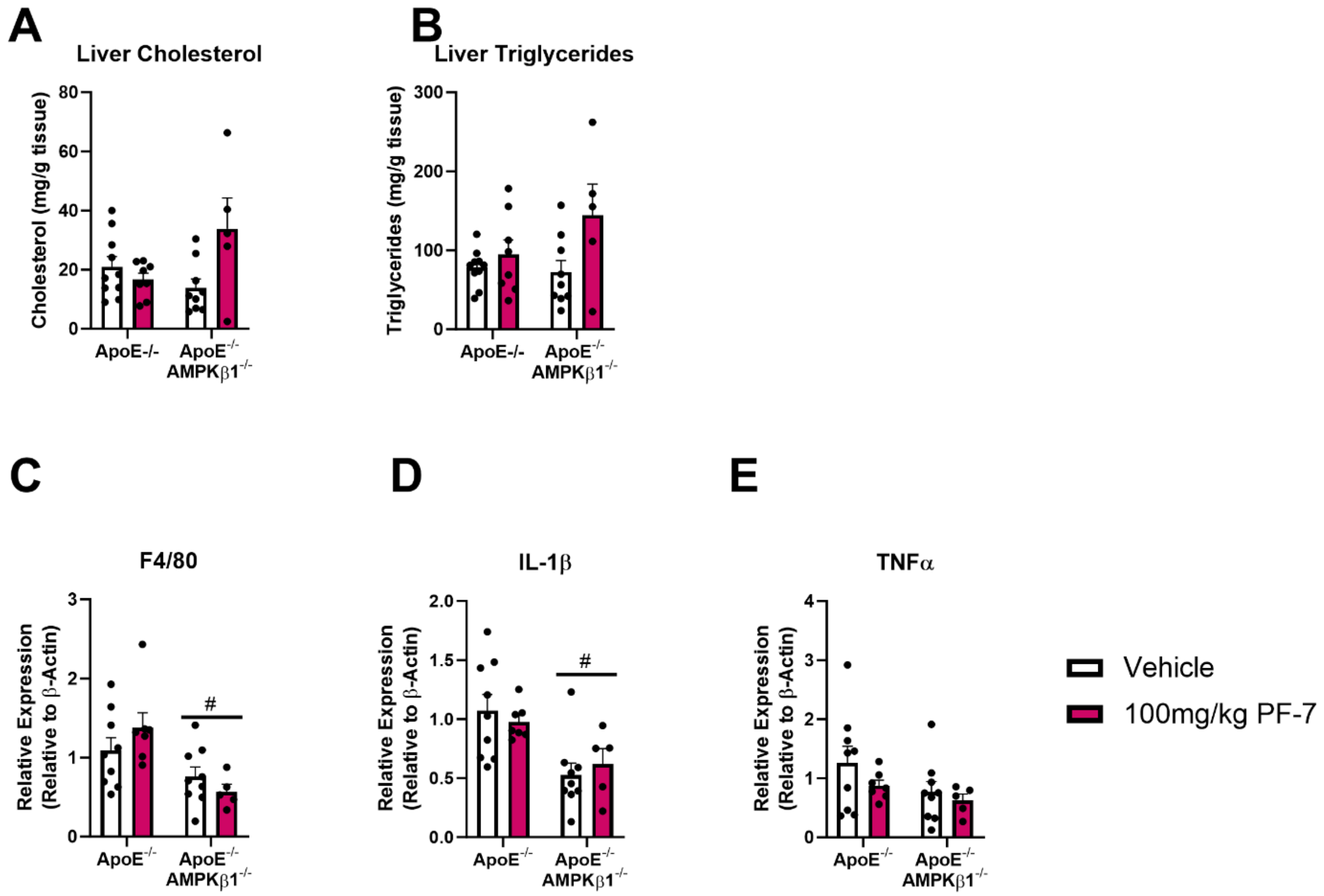

Supplemental Figure 2: PF-06409577 treatment does not alter hepatic lipids or inflammation. Related to Figure 1. ApoE$^{-/-}$ and ApoE$^{-/-}$ AMPK β1$^{-/-}$ mice were fed a Western diet and treated with 100mg/kg PF-06409577 for 6 weeks. **A,B**) hepatic cholesterol and triglcerides were measured, , **C,D,E**) hepatic inflammation was assessed by qPCR. # indicates $p<0.05$ for overall effect of genotype by two-way ANOVA.

Supplemental Figure 3

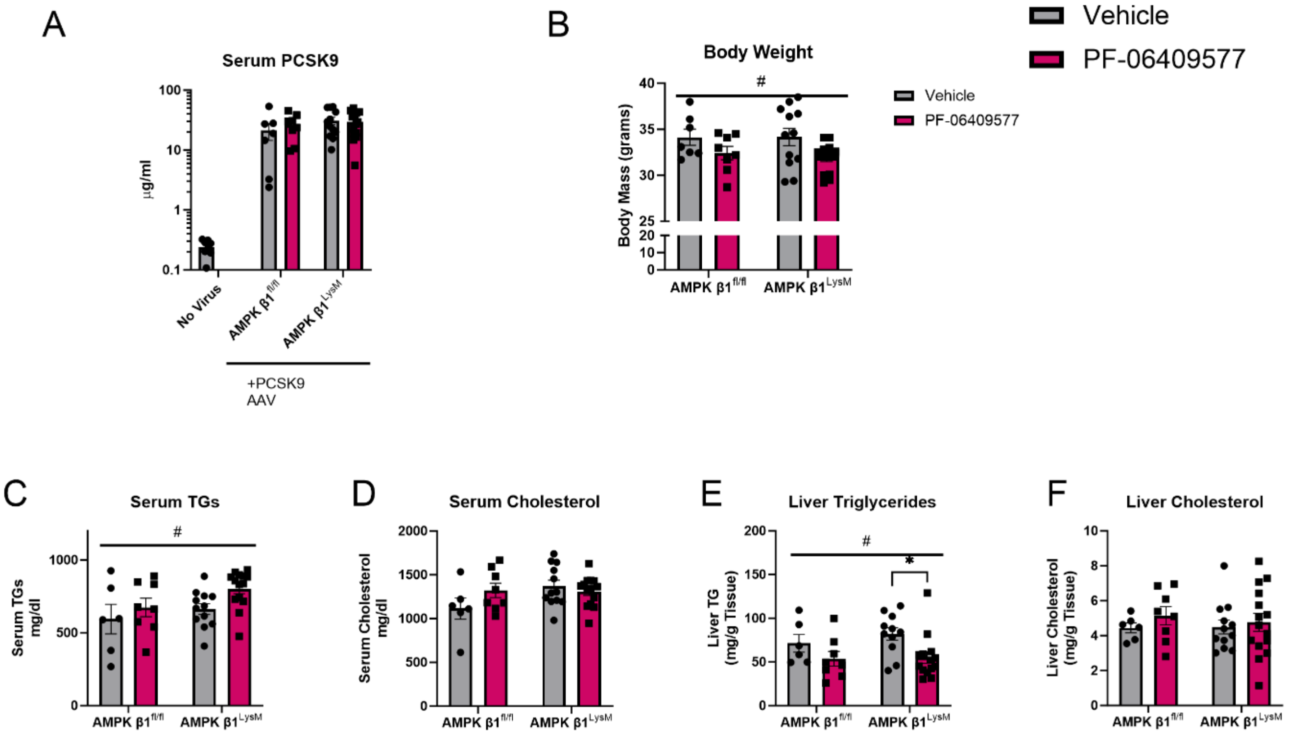

Supplemental Figure 3: PF-06409577 treatment does not alter serum or hepatic lipids in PCSK9 AAV injected mice. Related to Figure 2. AMPK $\beta1^{fl/fl}$ and AMPK $\beta1^{LysM}$ mice were injected (via the tail vein) with PSCK9 AAV. **A**) serum PCSK9 levels 1 week after injection with AAV. AMPK $\beta1^{fl/fl}$ and AMPK $\beta1^{LysM}$ mice were fed Western diet and treated with Vehicle or 100mg/kg PF-06409577 for 6 weeks. **B**) body weight, **C**) serum triglycerides, **D**) serum cholesterol, **E**) liver triglycerides, and **F**) liver cholesterol were measured. Data are presented as mean +/- s.e.m., # indicates p<0.05 for overall treatment effect by two-way ANOVA. * indicates p<0.05 by Sidaks post-hoc testing.
